# Cost-effectiveness of drug-eluting stents versus bare-metal stents in patients undergoing percutaneous coronary intervention

**DOI:** 10.1136/openhrt-2016-000445

**Published:** 2016-08-25

**Authors:** Louise Baschet, Sandrine Bourguignon, Sébastien Marque, Isabelle Durand-Zaleski, Emmanuel Teiger, Fanny Wilquin, Karine Levesque

**Affiliations:** 1 Capionis, Paris, France; 2 Stratégique Santé, Paris, France; 3 AP-HP Public Health, Henri Mondor Hospital, ECEVE-UMR1123—INSERM & UPEC, Paris, France; 4 AP-HP Public Health, Henri Mondor Hospital, Cardiovascular Department and INSERM U955, Creteil, France; 5 SNITEM, Courbevoie, France

**Keywords:** CORONARY ARTERY DISEASE, economic evaluation, drug-eluting stents, cost-effectiveness model

## Abstract

**Objective:**

To determine the cost-effectiveness of drug-eluting stents (DES) compared with bare-metal stents (BMS) in patients requiring a percutaneous coronary intervention in France, using a recent meta-analysis including second-generation DES.

**Methods:**

A cost-effectiveness analysis was performed in the French National Health Insurance setting. Effectiveness settings were taken from a meta-analysis of 117 762 patient-years with 76 randomised trials. The main effectiveness criterion was major cardiac event-free survival. Effectiveness and costs were modelled over a 5-year horizon using a three-state Markov model. Incremental cost-effectiveness ratios and a cost-effectiveness acceptability curve were calculated for a range of thresholds for willingness to pay per year without major cardiac event gain. Deterministic and probabilistic sensitivity analyses were performed.

**Results:**

Base case results demonstrated that DES are dominant over BMS, with an increase in event-free survival and a cost-reduction of €184, primarily due to a diminution of second revascularisations, and an absence of myocardial infarction and stent thrombosis. These results are robust for uncertainty on one-way deterministic and probabilistic sensitivity analyses. Using a cost-effectiveness threshold of €7000 per major cardiac event-free year gained, DES has a >95% probability of being cost-effective versus BMS.

**Conclusions:**

Following DES price decrease, new-generation DES development and taking into account recent meta-analyses results, the DES can now be considered cost-effective regardless of selective indication in France, according to European recommendations.

**Key questions**

**What is already known about this subject?**

- Much debate exists over the risk–benefit and cost-effectiveness ratio of drug-eluting stents (DES) over bare-metal stents (BMS) in terms of safety and efficacy. While DES are widely used in percutaneous coronary interventions, France has one of the lowest rates of use in Europe. Costs of DES devices have come down in recent years and second-generation DES development raises a need for evaluation of cost-effectiveness of DES versus BMS.

**What does this study add?**

- A cost-effectiveness analysis comparing BMS with DES including second-generation devices showed that in the French-specific setting, DES are associated with a robust cost-reduction mainly due to a reduction in second revascularisations, myocardial infarctions and stent thrombosis. No differences in overall survival were predicted.

**How might this impact on clinical practice?**

- Consider wider implementation of second-generation DES in percutaneous coronary interventions of this procedure in France.

## Introduction

Coronary artery disease is by far the most common cause of heart disease, resulting from the narrowing of coronary arteries (stenosis) caused by deposition of atherosclerotic plaque. A critical reduction of the blood supply to the heart may result in myocardial infarction or death.^1^ Coronary artery bypass grafting was the standard of care before the emergence of bare-metal coronary stents (BMS). Stents are placed via balloon dilation during a percutaneous coronary intervention (PCI). Nonetheless, restenosis occurs in a significant proportion of these patients with BMS (16–44%).^2^

Coronary drug-eluting stents (DES) first became available in 2000. They release antiproliferative and anti-inflammatory substances locally, inhibiting the proliferation of smooth muscle cells, thereby minimising a key aspect contributing to restenosis. In addition, the use of antiplatelet drugs and other therapeutic strategies to prevent thrombosis has improved long-term outcomes in these patients. DES are widely employed, with usage varying by country; in the USA, over 70% of PCI performed in 2006 used DES,^3^ while in France in 2007 DES were used in 42% of PCIs at a total cost of €106 million.

The relative safety of DES and BMS, notably with respect to stent thrombosis, is still under debate.^4^ First-generation DES, sirolimus-eluting stents and paclitaxel-eluting stents have been associated with an increased risk of late stent thrombosis when antiplatelet agents are withheld.^5^ ^6^ The introduction of second-generation DES, including everolimus-eluting stents and zotarolimus-eluting stents, has led to claims of improved safety with non-inferior efficacy compared with the first-generation stents, supported by numerous clinical trials.

In 2012, Palmerini *et al*^4^ published a large meta-analysis of 49 trials including 50 844 patients comparing the incidence of stent thrombosis with DES versus BMS. They conclude that some DES reduce risk of stent thrombosis, and none have shown a significant increase. The same year, Bangalore *et al*^7^ published a meta-analysis comparing the two stent types in terms of stent thrombosis, target vessel revascularisation, death and myocardial infarction with 117 762 patient-years of follow-up from 76 international randomised trials. While they found that the risk of death was not significantly different between the two stent types (long-term median rate from 11.5 per 1000 patient-years of follow-up with Zotarolimus-R to 16.6 with BMS^7^), there was nonetheless a lower risk on short-term outcomes (ORs from 0.55 to 0.67 at 1-year, median rate was 2.85% with BMS) and on long-term outcomes (risk ratio from 0.63 to 0.82, median rate 26.51 per 1000 patients-year with BMS) except for the paclitaxel-eluting stent. These updates of safety and efficacy of DES versus BMS are discussed in depth in the 2014 European Society of Cardiology (ESC)/the European Association for Cardio-Thoracic Surgery (EACTS) guidelines on myocardial revascularisation.^8^

The reduction in cost from the first somewhat expensive DES (€2600) to the newest generation DES (∼€950), combined with improvements in safety, long-term coronary effects and medical care with antiplatelet treatments, have modified the risk–benefit ratio and the cost-effectiveness, as discussed by Barone-Rochette *et al*.^9^ The 2014 European guidelines recommend the use of DES over BMS in all cases.^8^ In France, the last evaluation of DES was performed in 2009 by the Healthcare Strategies Assessment Department.^10^ In the light of the publication of these two meta-analyses in 2012 along with the 2014 revised European recommendations, an update of cost-effectiveness evaluation of the different stent types in France, including second generation DES, was performed. This study evaluates the cost-effectiveness of DES compared with BMS in patients undergoing PCI using the recent meta-analyses including second-generation DES.

## Methods

### Model structure

**Figure 1:**
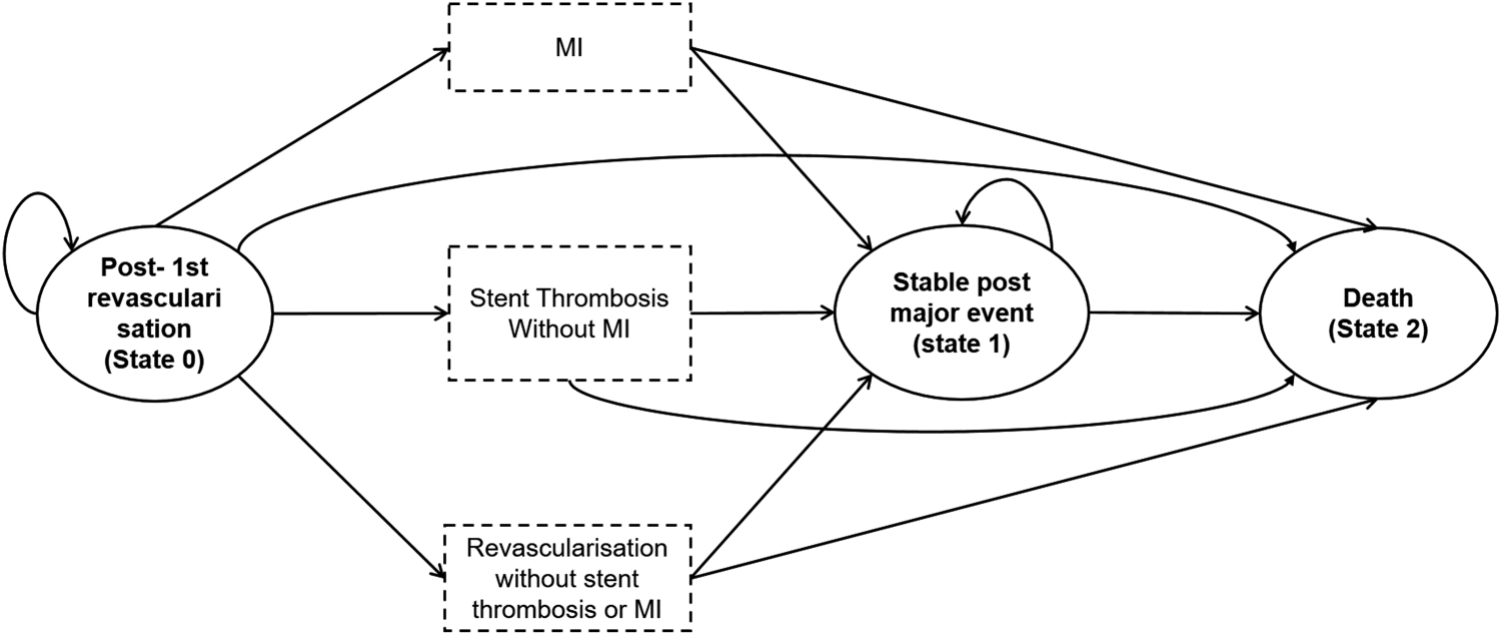
Markov model scheme. Patients start (state 0) with uncomplicated PCI revascularisation; then cycle between health states until death occurs or the 5-year period ends. Dotted boxes are events that cause a change of state. Each cycle is 6 months. The health states are equivalent for BMS and DES; however, probabilities and initial costs vary with the strategy. BMS, bare-metal stents; DES, drug-eluting stents; MI, myocardial infarction; PCI, percutaneous coronary intervention.

On the basis of the recent literature, and notably Bangalore *et al*'s^7^ meta-analysis, the model was Markov's model with three health states: major cardiac event-free state (state 0), post major cardiac event state (state 1) and death (state 2), as illustrated in figure 1. The three major events that lead to transition from state 0 to state 1 are: myocardial infarction, stent thrombosis and revascularisation. Only the first occurrence of any of these events was included in the model. We assumed that patients experiencing an event were either stable or dead at the following cycle; patients may also die directly from any state, without the listed events. This model is non-homogeneous and therefore probabilities of transition depend on the duration from the entry state in the model: first year and after 1 year.

The cycle length used was 6 months. Based on published efficacy studies,^7^ the most reliable time horizon is 5 years, because follow-up is between 2 and 4 years and therefore extrapolation over 5 years would introduce uncertainty. Half-cycle correction was applied as recommended by International Society for Pharmacoeconomics and Outcomes Research (ISPOR).^11^ Outcomes were estimated for the two cohorts undergoing PCI with either BMS or DES. The different kinds of DES were pooled since no difference was expected between kinds of DES. An annual discount rate of 4% was applied to costs and outcomes, according to recommendations of the Department of Economics and Public Health Assessment (French health technology assessment body).^12^

The cost-effectiveness of DES is expressed in terms of the incremental cost per year gained without a major cardiac event (myocardial infarction, stent thrombosis or revascularisation), in the context of the French National Health Insurance. A cost-utility approach was excluded because of lack of utility values in the French context for this pathology. The incremental cost per year gained was not considered because no difference in mortality was expected between the two strategies; other authors have assessed the efficiency of cardiac interventions with a cost per major adverse cardiovascular and cerebrovascular events (MACCE) averted, thus establishing a benchmark for our analyses.^13–15^

The base case patient cohort had a mean age of 60 years, and 60% of patients were men, concordant with Bangalore *et al*'s publication.^7^ The number of stents for PCI is assumed to be 1.5 per procedure, assuming that it does not depend on the choice of the stent, but only on the characteristics of the patient and lesions. The French national hospital database (PMSI) from 2007 reported a mean of 1.1 stents per DES PCI and 1.5 per BMS PCI.^16^ Our assumption is then conservative.

The model was developed with Microsoft Excel 2013 and Microsoft Visual Basic V.7.1 (Microsoft Corporation, Redmond, Washington, USA).

### Transition probabilities

The majority of event probabilities were extracted from Bangalore *et al*,^7^ and are summarised in table 1 according to the short term (1-year) and long term (after 1-year). Bangalore *et al* estimated rates for BMS and for each DES separately. To model the whole category of DES, the means of rates were used (eg, the rate of ‘definite or probable’ thrombosis in the short term (up to 1-year) for patients with BMS was estimated as 0.12%, varying from 0.04% for everolimus to 0.12% for zotarolimus, which were summarised by the mean 0.084%). Results from Palmerini *et al*^4^ were also used for definite or probable stent thrombosis in sensitivity analyses; OR for DES versus BMS varied from 0.34 for cobalt-chromium everolimus DES to 1.13 for phosphorylcholine polymer-based zotarolimus DES, summarised by a mean 0.61. This mean OR applied to a base rate of 0.12% from Bangalore leads to a 1-year rate in the DES group of 0.073%. The two sources are then consistent.

**Table 1:** Summary of events and transition probabilities

From	To	Cohort	Cycles	6-month probabilities			Source
				Base case	Minimum	Maximum	
Major cardiac event-free state	Myocardial infarction	BMS	First year	2.14%	1.83%	2.48%	(7)
			Following years	1.32%	1.16%	1.48%	(7)
		DES	First year	1.40%	0.82%	2.19%	(7)
			Following years	1.02%	0.61%	1.57%	(7)
Major cardiac event-free state	Stent thrombosis	BMS	First year	0.06%	0.00%	0.60%	(7)
			Following years	0.26%	0.11%	5.20%	(7)
		DES	First year	0.042%	0.00%	0.70%	(7)
			Following years	0.26%	0.11%	5.50%	(7)
Major cardiac event-free state	Second revascularisation	BMS	First year	8.22%	7.17%	9.15%	(7)
			Following years	4.37%	4.06%	4.35%	(7)
		DES	First year	2.88%	1.33%	5.31%	(7)
			Following years	2.15%	1.22%	3.38%	(7)
Myocardial infarction	Death	−	−	3.20%	2.90%	3.50%	(17)
Stent thrombosis	Death	−	−	1.00%	0.50%	20.00%	Calibration
Second revascularisation	Death	−	−	1.00%	0.50%	20.00%	Calibration
Stabilised post major cardiac event state	Death	−	−	1.01%	0.50%	10.00%	(17)
Global death		BMS	First year	0.12%	0.00%	0.78%	(7)
			Following years	0.83%	0.65%	1.07%	(7)
		DES	First year	0.11%	0.00%	1.02%	(7)
			Following years	0.71%	0.25%	1.10%	(7)

BMS, bare-metal stents; DES, drug-eluting stents.

No specific mortality probabilities during each cardiac event and from each health state were available for this specific population of patients after a first PCI. Thus, only global mortality for all causes was considered. Mortality probability from the post major cardiac event state was based on a randomised trial published by Kastrati *et al*^17^ evaluating recurrences in patients with coronary in-stent restenosis, assuming a similar risk for myocardial infarction, stent thrombosis and new revascularisation. Death probability during the 6 months following a myocardial infarction was estimated from the Swedish Coronary Angiography and Angioplasty Registry (SCAAR) registry, as in the model proposed by Wisløff *et al*.^18^

We assume that PCI for patients free from any of the three listed major cardiac events did not lead to increased mortality risk compared with the French general population at the same age and same sex proportion. Other mortality probabilities were estimated empirically by calibration to be coherent with global mortality estimated in Bangalore *et al*'s meta-analysis. Since cohorts grow older during a cycle, mortality probabilities are assessed to never be lower than in the general population. Base mortality probabilities were estimated using the French National Institute of Statistics and Economic Studies (INSEE). In the absence of the Kaplan-Meier estimation and to simplify, exponential function was used to convert the annual rate to 6 month probabilities and to extrapolate until time horizon.

### Costs

Costs were derived from the perspective of the French National Health Insurance, as evaluated in 2012, and only direct medical costs were used. All hospital costs were taken from the PMSI, and diagnosis-related groups were used to identify hospital stays for DES and BMS PCIs, taking into account stay duration and additional costs for intensive care. Stent device prices were added to the cost of the initial hospital stay for PCI, including the number of implanted stents.

Standard care after the first revascularisation, mainly involving patient monitoring, included drug treatment costs and the costs of radiology and laboratory tests, according to the recommendations for antiplatelet treatment of coronary disease from the French National Insurance.^19^ Follow-up was estimated from the Haute Autorité de Santé (HAS) guideline for coronary heart disease. Treatment costs were distinguished for myocardial infarction, bypass surgery or second PCI. Costs of the events related to hospitalisation were taken from the PMSI extraction in 2012. Cost of death was based on a specific diagnosis-related group. Resource quantities and unit costs are summarised in table 2.

**Table 2:** Summary of costs

	Inpatient	Outpatient	Base case	Minimum	Maximum	SD
Initial revascularisation cost (€)						
BMS medical care	2957.42	154.58	3112	1841.93	7042.57	1505.69
BMS device	550		550	440	550	
DES medical care	2957.42	218.75	3176.17	1893.27	7119.57	1505.69
DES device	925		925	740	925	
Following cycles standard care cost per cycle in major cardiac event-free state or post major cardiac event state		132.73	132.73	106.18	159.27	26.55
Cost per event (€)						
Myocardial infarction	4190.23	132.73	4322.96	681.23	12956.31	3452.94
Stent thrombosis	2547.68	148.97	2696.65	398.96	11485.83	3273.25
Second revascularisation	4289.53	141.23	4430.76	688.04	5568.88	4175.21
Death	822.54		822.54	658.03	987.05	164.51

Cycle length is 6 months.

BMS, bare-metal stents; DES, drug-eluting stents.

### Sensitivity analyses

One-way sensitivity analyses on parameter values and structural assumptions were undertaken to assess the robustness of results to changes in individual model parameters while the remaining assumptions were held constant.^20^ In terms of demographics, the initial age varied from 40 to 70 years, sex ratios varied from 45% to 75%, and number of stents from 1.1 to 2.7, according to range values in previous models. The discount rate varied to 0%, 2.5% and 6.0%.

Assumption of a constant mortality rate after PCI for patients free from cardiac events was evaluated by considering a risk ratio of 2 for this population, compared with the general population. Each transition probability varied between the credibility limits. For the DES cohort, to consider the uncertainty of pooled stents, the most extreme lowest and upper limits of all credibility limits were used. This approach, considering the broadest range of values, is thus a ‘worst case’ scenario. For parameters chosen by calibration, extreme tested values were 0.5 and 20%. Costs variations are described in table 2 and were derived from the 95% CI from the database extraction. For calibration analysis, results were evaluated over a lifetime horizon, to validate what may happen after 5 years, and the expected life expectancy was used.

Probabilistic sensitivity analysis (PSA) was used to assess overall parameter uncertainty in the model. Point estimates for each parameter were replaced with values sampled from statistical distributions and the incremental cost-effectiveness ratio (ICER) was recalculated using the new resampled values.^20^ Probabilities followed β distribution and costs γ distribution (see online supplementary appendix 1 for more details). This process was repeated 2000 times to estimate uncertainty and to predict the likelihood that DES would be cost-effective at different cost-effectiveness thresholds (the value the decision-maker is willing to pay for each additional cardiac event-free year).

10.1136/openhrt-2016-000445.supp1 supplementary appendix

The uncertainty related to the structure of the model is not explored, particularly on the choice to model only the first major cardiac event occurrence, because of lack of data, and because the main outcome is not influenced by what happens after this first event.

## Results

### Base case

Base case results are presented in table 3, including the proportion of patients with each event. Over 5 years, after a discount, DES was expected to improve major cardiac event-free survival duration by 0.541 years (more than 6 months) compared with BMS. The model predicted that, over 5 years, the cost decreases by €184. This saving is mainly due to the avoided second revascularisation (19.0% over 5 years in the DES cohort compared with 37.1% in the BMS cohort). In the base case, DES dominate over BMS.

**Table 3:** Base case deterministic outcomes for DES versus BMS

	BMS	DES	Delta
Time (years) in health state			
Major cardiac event-free	3.382	4.077	0.695
Stabilised post major cardiac event	1.462	0.790	−0.672
LY	4.844	4.867	0.022
5-year proportion of patients with transition*			
MI	10.8%	9.2%	−1.6%
ST (w/o MI)	1.9%	1.7%	−0.2%
Revascularisation (w/o MI or ST)	37.1%	19.0%	−18.1%
Cumulative cost (€)**	7 153.71 €	6 969.86 €	−183.85 €
Major cardiac event-free survival years (discount)**	2.908	3.448	0.541
Cost per major cardiac event-free survival year gained (discount)**			−340.13 €

*Only the first event occurrence is considered.

**Over 5 years.

BMS, bare-metal stent; DES, drug-eluting stent; LY, Life-year; MI, myocardial infarction; ST, stent thrombosis; w/o, without.

### Deterministic sensitivity analyses

**Figure 2:**
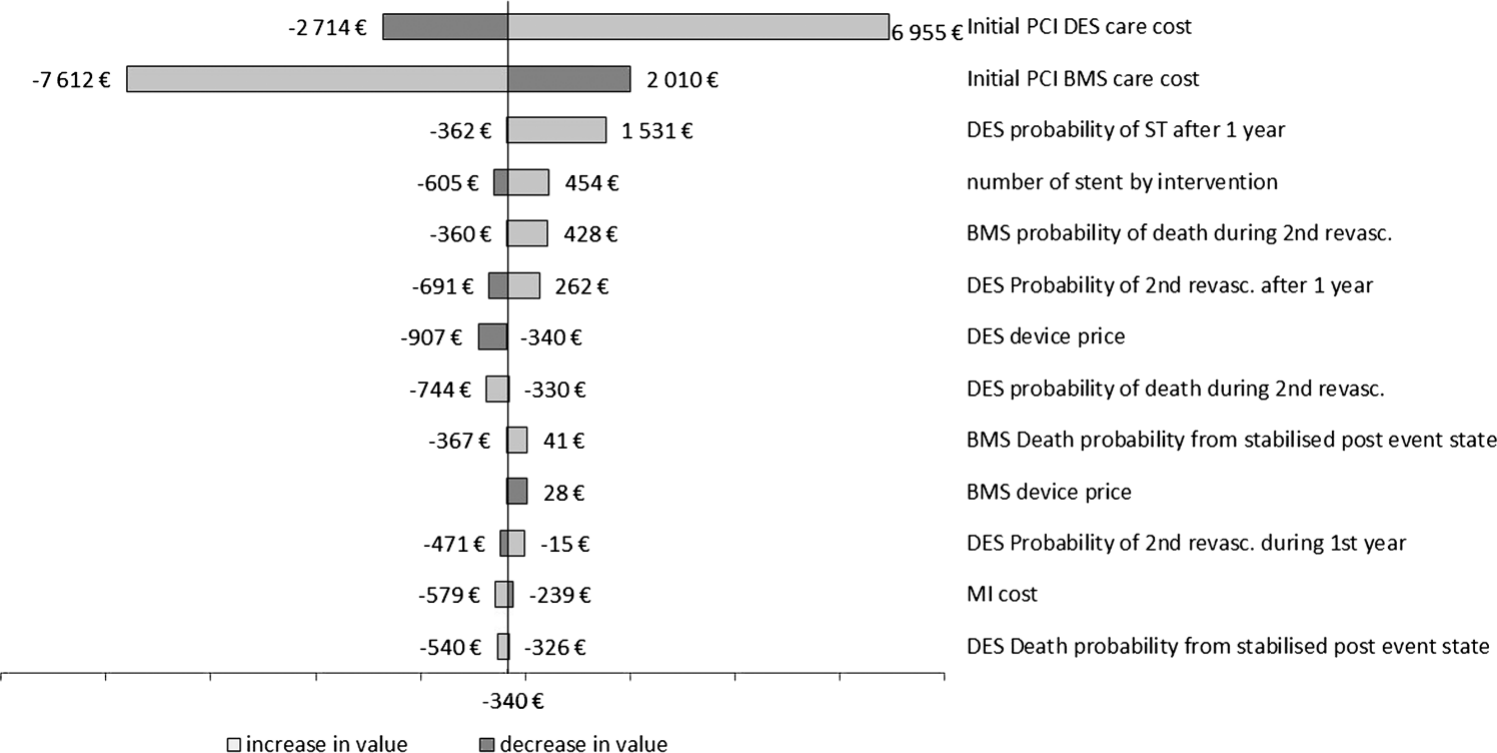
Tornado diagram of one-way deterministic sensitivity analysis. Numbers are the estimated ICER when changing parameter value to a maximal value (light grey) or a minimal value (dark grey). Only parameters giving variation above €200 are shown. BMS, bare-metal stent; DES, drug-eluting stent; ICER, incremental cost-effectiveness ratio; MI, myocardial infarction; PCI, percutaneous coronary intervention; ST, stent thrombosis.

A tornado diagram of the one-way deterministic analyses shows the effect on the estimated ICER if one model assumption is altered while other assumptions remain at base case values (figure 2). Only parameters that lead to a variation >€200 on ICER are shown. In particular, if the number of stents varied from 1.1 to 2.7, ICER varied from −€605 to €454. The two most influential parameters are the cost for initial PCI for both strategies. The uncertainty comes from the PMSI database extraction variability. The worst case is an initial PCI cost (for BMS) fixed at €7120 instead of €3176, leading to an overall incremental cost of €3760, and an ICER of €6955. It can thus be concluded that results are robust to uncertainty.

On the lifetime horizon, for calibration, life expectancy was 23.1 years in the BMS group and 23.5 years in the DES group. There was no difference in mortality with BMS versus DES, and our model shows no differences in overall survival between the two strategies (30.6% vs 29.3% at 20 years). DES was expected to improve major cardiac event-free survival by 2.6 years more than BMS, with a decreased cost of €1. DES dominated BMS in all settings; however, these results may not be reliable in terms of long-term extrapolation.

### Probabilistic sensitivity analyses

**Figure 3:**
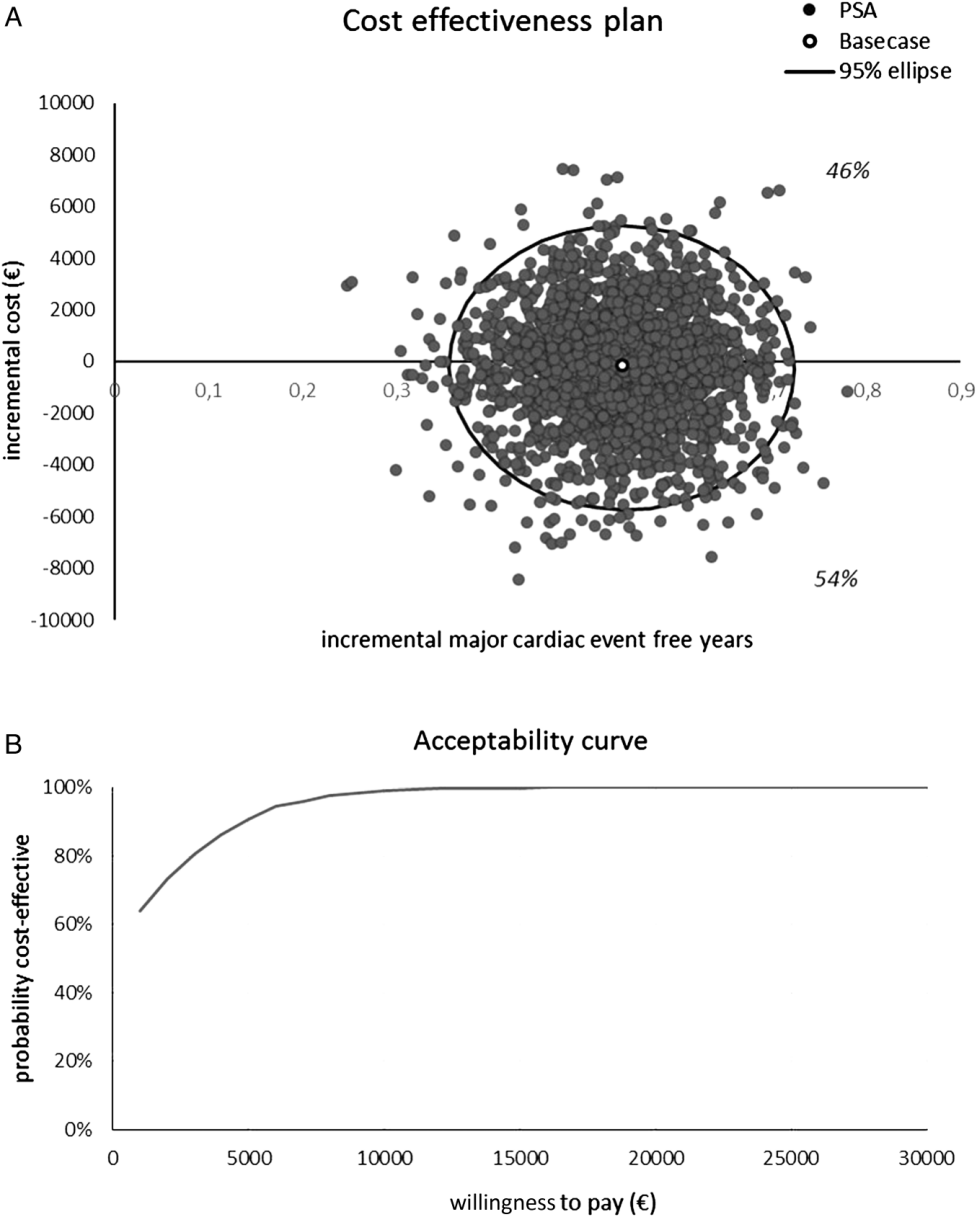
Sensitivity analysis results. (A) Cost-effectiveness plan and (B) acceptability curve. PSA, Probabilistic sensitivity analysis.

The spread of simulated points is shown in figure 3A and the acceptability curve in figure 3B. Fifty-four per cent of simulations show that DES are dominant, and 46% that DES are more expensive and more effective. Probabilistic sensitivity analysis indicates that using a cost-effectiveness threshold of €7000 per major cardiac event-free year gained, DES has a >95% probability of being cost-effective versus BMS, and with a threshold of €3000, this probability is more than 80%. We conclude that the model is robust to uncertainty.

## Discussion

Controversy exists over the cost-effectiveness of DES compared with BMS for patients undergoing PCI. In two systematic reviews published in 2007, 10 studies were discussed by Hill *et al*^21^ and 13 studies by Kuukasjärvi *et al*.^22^ The diversity of conclusions may be explained by the multiplicity of designs for such evaluations, each design associated with its own biases: ancillary studies of randomised trials, direct comparison in a single-centre; model-based evaluations, and data from patient registries.^9^ Nonetheless, most of them conclude that DES can be cost-effective for high-risk subgroups. Another factor to take into account is that most of these studies used results from the first-generation DES.^9^ ^23^ In 2011, Müller-Riemenschneider *et al*^24^ asserted the need for rigorous economic evaluations using second-generation DES. Until now, models used do not take into account the stent thrombosis event.^18^

Our current analysis, which includes second-generation DES, reports promising results which are consistent with a recent study published by Wisløff *et al*,^18^ using randomised controlled trials, and Swedish registry analyses in secondary analysis.

In addition, Bangalore *et al*'s meta-analysis is the largest and the most recent data source that we could use.

DES was better than BMS in terms of cost-reduction (€184 saving with the base case), attributed largely to a reduction in second revascularisations, and the absence of myocardial infarctions and stent thrombosis. Results were consistent under sensitivity analyses.

### Limitations

Our Markov model is based on results from randomised clinical trials. Registry data may be considered as more appropriate to estimate a real-life effect. This is particularly important given that in randomised clinical trials, second revascularisation is frequently identified from complete investigations, and can be identified in asymptomatic patients (so-called ‘protocol-driven’ reintervention), in comparison with real life, where second revascularisation is usually identified on the basis of clinical symptoms.^1^ To investigate this uncertainty, we added an exploratory analysis, using probabilities from the Swedish SCAAR registry, as proposed by Wisløff et al^18^ and Palmerini *et al*^4^ results for stent thrombosis. The results were very encouraging, with an ICER at €7823, and using a cost-effectiveness threshold of €27 000 per major cardiac event year gained, DES has a >75% probability of being cost-effective versus BMS. The main difference with our initial method was the method for estimating necessity of revascularisation in randomised clinical trials with a rigorous follow-up with frequent imaging measurements, and less strict follow-up in real life (predicted proportions of 14.8% of second revascularisation at 5 years with BMS and 13.4% with DES).

With respect to uncertainty of probabilities chosen by calibration, for death after a second revascularisation, the assumed 1% is close to the non-rigorous but informative estimation of 0.8% from the PMSI database extraction. Sensitivity analyses showed that even extreme values had a small impact on results. A cost-utility analysis may be of interest; however, utilities associated with each health state, with a French specificity, should be investigated first.

### France in the European context

In France, since the publication by the HAS in 2009 of selected recommendations, for economic reasons DES are currently reserved first for patients with high risk of stenosis (diabetes, long lesion, small vessel). However, in 2014, the ESC/EACTS guidelines on myocardial revascularisation published indications for new-generation DES: increased efficacy and safety of new-generation DES have enabled their unrestricted use in patients with coronary artery disease requiring PCI, including patients with diabetes, multivessel and left main coronary artery disease, acute myocardial infarction, saphenous vein graft and restenotic lesions, and chronic total occlusions.^8^ New-generation DES should therefore be considered the default option in all clinical settings and lesion subsets.

In France, according to the healthcare products pricing committee 2013 activity report, more than 196 000 stents were implanted in 2013, and 72.5% were DES, the lowest proportion in Europe, with 89% in the UK/Ireland, 78% in Italy, 77% in Germany and 74% in Spain (Eucomed data).

### Conclusion

In the light of the decreasing costs of the DES device, the development of new-generation devices and outcomes of recent meta-analyses, the DES can now be considered cost-effective irrespective of any restrictions for selective indications according to the 2014 European recommendations. This supports the need for discussion of the use of DES in France.

## References

[1] Guidance on the use of coronary artery stents | Guidance and guidelines | NICE [Internet]. (cited 8 Apr 2015). https://www.nice.org.uk/guidance/ta71

[2] Duckers HJ, Nabel EG, Serruys PW, eds. Essentials of restenosis [Internet]. Totowa, NJ: Humana Press, 2007 (cited 20 Jun 2016). http://link.springer.com/10.1007/978-1-59745-001-0

[3] Eisenberg MJ Drug-eluting stents: the price is not right. Circulation 2006;114:1745–54; discussion 1754. 10.1161/CIRCULATIONAHA.106.646190 17043178

[4] Palmerini T, Biondi-Zoccai G, Della Riva D Stent thrombosis with drug-eluting and bare-metal stents: evidence from a comprehensive network meta-analysis. Lancet 2012;379:1393–402. 10.1016/S0140-6736(12)60324-9 22445239

[5] Pfisterer M, Brunner-La Rocca HP, Buser PT Late clinical events after clopidogrel discontinuation may limit the benefit of drug-eluting stents: an observational study of drug-eluting versus bare-metal stents. J Am Coll Cardiol 2006;48:2584–91. 10.1016/j.jacc.2006.10.026 17174201

[6] Airoldi F, Colombo A, Morici N Incidence and predictors of drug-eluting stent thrombosis during and after discontinuation of thienopyridine treatment. Circulation 2007;116:745–54. 10.1161/CIRCULATIONAHA.106.686048 17664375

[7] Bangalore S, Kumar S, Fusaro M Short- and long-term outcomes with drug-eluting and bare-metal coronary stents: a mixed-treatment comparison analysis of 117 762 patient-years of follow-up from randomized trials. Circulation 2012;125:2873–91. 10.1161/CIRCULATIONAHA.112.097014 22586281

[8] Kolh P, Windecker S, Alfonso F 2014 ESC/EACTS Guidelines on myocardial revascularization: the Task Force on Myocardial Revascularization of the European Society of Cardiology (ESC) and the European Association for Cardio-Thoracic Surgery (EACTS). Developed with the special contribution of the European Association of Percutaneous Cardiovascular Interventions (EAPCI). Eur J Cardiothorac Surg 2014;46:517–92. 10.1093/ejcts/ezu366 25173601

[9] Barone-Rochette G, Machecourt J, Vanzetto G The favorable price evolution between bare metal stents and drug eluting stents increases the cost effectiveness of drug eluting stents. Int J Cardiol 2013;168:1466–71. 10.1016/j.ijcard.2012.12.054 23336951

[10] Haute Autorité de Santé. Assessment of drug-eluting stents [Internet] 2009 (cited 7 Aug 2015). http://www.has-sante.fr/portail/upload/docs/application/pdf/2009-11/assessment_of_drug-eluting_stents.pdf 10.1016/j.rmr.2012.09.015 23200597

[11] Siebert U, Alagoz O, Bayoumi AM State-transition modeling: a report of the ISPOR-SMDM modeling good research practices task force-3. Value Health 2012;15:812–20. 10.1016/j.jval.2012.06.014 22999130

[12] Department of Economics and Public Health Assessment. A methodological guide. Choices in methods for economic evaluation [Internet] 2012 http://www.has-sante.fr/portail/upload/docs/application/pdf/2012-10/choices_in_methods_for_economic_evaluation.pdf

[13] Greenhalgh J, Hockenhull J, Rao N Drug-eluting stents versus bare metal stents for angina or acute coronary syndromes. Cochrane Database Syst Rev 2010 (5):CD004587 10.1002/14651858.CD004587.pub2 20464732

[14] Dijksman LM, Hirsch A, Windhausen F Cost-effectiveness of early versus selectively invasive strategy in patients with acute coronary syndromes without ST-segment elevation. Int J Cardiol 2009;131:204–11. 10.1016/j.ijcard.2007.10.019 18199496

[15] Brunner-La Rocca HP, Kaiser C, Bernheim A Cost-effectiveness of drug-eluting stents in patients at high or low risk of major cardiac events in the Basel Stent KostenEffektivitäts Trial (BASKET): an 18-month analysis. Lancet Lond Engl 2007;370:1552–9. 10.1016/S0140-6736(07)61660-2 17980734

[16] Tu JV, Bowen J, Chiu M Effectiveness and safety of drug-eluting stents in Ontario. N Engl J Med 2007;357:1393–402. 10.1056/NEJMoa071076 17914040

[17] Kastrati A, Mehilli J, von Beckerath N Sirolimus-eluting stent or paclitaxel-eluting stent vs balloon angioplasty for prevention of recurrences in patients with coronary in-stent restenosis: a randomized controlled trial. JAMA 2005;293:165–71. 10.1001/jama.293.2.165 15644543

[18] Wisløff T, Atar D, Sønbø Kristiansen I Cost effectiveness of drug-eluting stents as compared with bare metal stents in patients with coronary artery disease. Am J Ther 2013;20:596–601. 10.1097/MJT.0b013e3182211a01 21822114

[19] Assurance maladie. Antiagrégants plaquettaires—Traitement d'entretien de la maladie coronaire [Internet] 2013 (cited 7 Aug 2015). http://www.ameli.fr/fileadmin/user_upload/documents/1112013_memo_aap_bdef.pdf

[20] Briggs A, Sculpher M, Claxton K Decision modelling for health economic evaluation. Oxford University Press, 2006:250 p.

[21] Hill RA, Boland A, Dickson R Drug-eluting stents: a systematic review and economic evaluation. Health Technol Assess 2007;11:iii, xi–221. 10.3310/hta11460 17999841

[22] Kuukasjärvi P, Räsänen P, Malmivaara A Economic evaluation of drug-eluting stents: a systematic literature review and model-based cost–utility analysis. Int J Technol Assess Health Care 2007;23:473–9. 10.1017/S0266462307070560 17937836

[23] Canoui-Poitrine F, Jeanblanc G, Alberti C Cost effectiveness of sirolimus-eluting stents compared with bare metal stents in acute myocardial infarction: insights from the TYPHOON trial. Appl Health Econ Health Policy 2009;7:19–29. 10.1007/BF03256139 19558192

[24] Müller-Riemenschneider F, Reinhold T, Willich SN [Second-generation DES: new, but also cost-effective?]. Herz 2011;36:254–61. 10.1007/s00059-011-3463-2 21509580

