# Supplementary material for: Cost-effectiveness of drug-eluting stents versus bare-metal stents in patients undergoing percutaneous coronary intervention
Source: Open Heart. 2016 Aug 25;3(2):e000445. doi: 10.1136/openhrt-2016-000445 (PMC5013343; doi:10.1136/openhrt-2016-000445)
Supplement: supplementary appendix [file openhrt-2016-000445supp_appendix.pdf]

Supplementary material 1: parameters base-case, one-way sensitivity analyses, and probabilistic analyses

parameter	basecase	min	max	sd	distribution PSA	parameters PSA	
**population parameters**							
Age at baseline	60	40	70	8	Normal	μ=60	σ=8
% male	60%	0,45	0,75	0,1	Beta	α=13,8	β=9,2
Number of stents per intervention	1,5	1,1	2,7	0,5			
**Transition probabilities**							
BMS: Major cardiac event free state (year 1) -> Myocardial infarction	2,14%	0,00%	2,48%	0,50%	Beta	α=18,2	β=834,2
BMS: Major cardiac event free state (years >1) -> Myocardial infarction	1,32%	1,16%	1,48%	0,06%	Beta	α=428,7	β=32137,5
BMS: Major cardiac event free state (year 1) -> Stent thrombosis	0,06%	0,00%	0,60%	0,12%	Beta	α=0,3	β=419,8
BMS: Major cardiac event free state (years >1) -> Stent thrombosis	0,36%	0,28%	0,45%	0,03%	Beta	α=109,9	β=30597,0
BMS: Major cardiac event free state (year 1) -> Second revascularisation	8,22%	7,17%	9,15%	0,40%	Beta	α=396,7	β=4430,2
BMS: Major cardiac event free state (years >1) -> Second revascularisation	4,37%	4,06%	4,35%	0,06%	Beta	α=5389,5	β=117869,4
DES: Major cardiac event free state (year 1) -> Myocardial infarction	1,40%	0,82%	2,19%	0,28%	Beta	α=25,5	β=1796,6
DES: Major cardiac event free state (years >1) -> Myocardial infarction	1,02%	0,61%	1,57%	0,19%	Beta	α=27,9	β=2706,8
DES: Major cardiac event free state (year 1) -> Stent thrombosis	0,04%	0,00%	0,70%	0,14%	Beta	α=0,1	β=211,6
DES: Major cardiac event free state (years >1) -> Stent thrombosis	0,26%	0,11%	5,20%	1,02%	Beta	α=0,1	β=24,2
DES: Major cardiac event free state (year 1) -> Second revascularisation	2,88%	1,33%	5,31%	0,79%	Beta	α=12,8	β=429,7
DES: Major cardiac event free state (years >1) -> Second revascularisation	2,15%	1,22%	3,38%	0,43%	Beta	α=24,3	β=1104,2
Myocardial infarction -> Death	3,20%	2,90%	3,50%	0,12%	Beta	α=688,3	β=20821,8
Stent thrombosis -> Death	1,00%	0,50%	20,00%	3,90%	Beta	α=0,1	β=5,5
Second revascularisation -> Death	1,00%	0,50%	20,00%	3,90%	Beta	α=0,1	β=5,5
Stabilised post major cardiac event state -> Death	1,01%	0,50%	10,00%	1,90%	Beta	α=0,3	β=26,3
**Costs**							
BMS medical care Initial revascularisation cost (€)	3112	1841,9	7042,57	1505,7	Gamma	α=4,3	β=728,5
BMS device	550	440	550				
DES medical care Initial revascularisation cost (€)	3176,17	1893,3	7119,57	1505,7	Gamma	α=4,4	β=713,8
DES device	925	740	925				
Following cycles standard care cost per cycle in major cardiac event-free state or post major cardiac event state	132,73	106,18	159,27	26,55	Gamma	α=25,0	β=5,3
Myocardial infarction (€)	4322,96	681,23	12956,3	3452,9	Gamma	α=1,6	β=2758,0
Stent thrombosis (€)	2696,65	398,96	11485,8	3273,3	Gamma	α=0,7	β=3973,1
Second revascularisation (€)	4430,76	688,04	5568,88	4175,2	Gamma	α=1,1	β=3934,4
Death (€)	822,54	658,03	987,05	164,51	Gamma	α=25,0	β=32,9

BMS: Bare-metal stent ; DES: Drug-eluting stent

Probabilities followed beta distribution (parameters based on mean and variance : $\alpha = \mu\left(\frac{\mu(1-\mu)}{\sigma^2} - 1\right)$ *and* $\beta = \alpha\left(\frac{1}{\mu} - 1\right)$*) and costs gamma distribution (parameters based on mean and variance :* $\alpha = \frac{\mu^2}{\sigma^2}$ *and* $\beta = \frac{\sigma^2}{\mu}$*)*
